# Interface effects in hybrid hBN-graphene nanoribbons

**DOI:** 10.1038/s41598-019-39763-5

**Published:** 2019-03-05

**Authors:** Carlos Leon, Marcio Costa, Leonor Chico, Andrea Latgé

**Affiliations:** 1 Instituto de Física, Universidade Federal Fluminense, Av. Litorânea sn, 24210-340 Niterói-Rio de Janeiro, RJ Brazil; 2 Instituto de Ciencia de Materiales de Madrid (ICMM), Consejo Superior de Investigaciones Científicas (CSIC), Madrid, 28049 Spain

## Abstract

We analyze the electronic properties of a hybrid graphene-BN nanoribbon system, using a Hubbard model Hamiltonian within a mean field approximation. Due to the different electronegativities of the boron and nitrogen atoms, an electric field is induced across the zigzag graphene strip, breaking the spin degeneracy of the electronic band structure. Optimal tight-binding parameters are found from first-principles calculations. Edge potentials are proposed as corrections for the on-site energies, modeling the BN-graphene nanoribbon interfaces. We show that half-metallic responses in the hybrid systems may be driven with the help of an external electric field. We also study the role of defects across the graphene nanoribbon and at the h-BN/graphene interface regions. Modulations on the spin-dependent gaps may be achieved depending on the nature and position of the defect, constituting a way towards spin-gap engineering by means of spatial doping.

## Introduction

Graphene is a zero-band-gap semiconductor with valence and conduction bands touching at the corners of the Brillouin zone. However, in order to use graphene in semiconductor electronics, such as in field-effect transistors (FETs), it is very important to open a band gap. This can be achieved, for instance, by patterning graphene into quasi-one-dimensional nanoribbons or tuning its properties for its use as a two-dimensional semiconductor^1^. In nanoribbons, the opened gap depends on the edge type and the width of the strip^2–4^. Alternatively, combining the properties of large band gap semiconductors, such as hexagonal-boron nitride (h-BN), with the conductive properties of graphene, appears as a promising route for gap modulation^5^. In fact, h-BN has the same honeycomb lattice as graphene, but composed of alternating boron and nitrogen atoms with a lattice constant (2.50 Å) very similar to that of graphene (2.46 Å). The band gap of h-BN is [∼5–6]*eV*^6^. Hybrid graphene and h-BN materials have been synthesized using lithography patterning and sequential CVD growth steps^7^. Actually, efficient epitaxial growth routes of BN onto graphene edges are possible under particular experimental conditions^8^, allowing a good matching between both lattices.

Gap engineering is also possible by means of external electric fields^9–11^. Insulator-metal transitions in BN nanoribbons have been predicted with the application of transversal electric fields^12^; the actual critical field depends on the width of the ribbon. Particularly, when an electric field is applied in the transversal direction of zigzag graphene nanoribbons (ZGNRs), the system behaves as a half-metallic material^13,14^. Namely, the spin-degeneracy is broken and the energy gap for electrons with spin down (spin up) decreases (increases) until a critical electric field is reached, for which there is a null gap for the spin-down channel, and the electrons in the other channel have a direct band gap. This opens the possibility of exploring spin filtering properties of carbon systems^15^. Interestingly, such half-metallic behavior can be achieved even in the absence of an external electric field for some systems^16–19^ as embedded ZGNRs in zigzag h-BN nanoribbons (ZBNNRs)^20^.

Graphene/h-BN heterostructures have been proposed for engineering applications; for example, CO_2_ -capture devices^21^, electronic rectifiers^22^, and spin-filters activated by strain^23^, or by edge hydrogenation effects^24^. Induced half-metallic responses due to spin polarization through defective interfaces of h-BN/graphene was reported in the work of Lan *et al*.^25^. Also, laterally repeating h-BN/graphene systems present a variety of electronic properties according to the geometry of the h-BN/layers, and can be potentially used in optical devices^26^. The electronic properties can also be modified by dihydrogenation of the edges of hybrid C/BN nanostructures^27^, promoting interesting semiconductor-to-half-metal-to-metal transitions.

**Figure 1:**
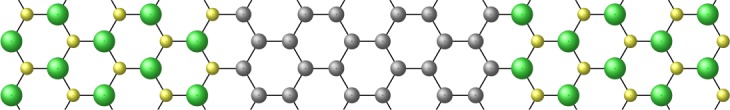
Portion of a zigzag nanoribbon hybrid system, 5BN/6 G/5BN, composed by two translational unit cells (armchain chains). The edges of the ribbon are parallel to the vertical dotted line. Green, yellow, and gray balls represent B, N and C atoms, respectively.

Our study focuses on hybrid systems of the form *m*-ZBNNR/*n*-ZGNR/*m*-ZBNNR (see Fig. 1), with *m* and *n* being the number of zigzag boron nitride and carbon chains, respectively. For the sake of simplicity, in what follows we label this hybrid system with zigzag edges and interfaces as *m* BN/*n* G/*m* BN. A tight-binding (TB) model with electronic correlation given by a Hubbard-like term is used to account for spin-dependent solutions. Simple TB calculations can reproduce quite well the electronic bands near the Fermi level for ZGNRs when compared to first-principles results^28^. For the case of ZBNNRs, more complex descriptions are necessary due to the large ionicity of BN.

Approaches to overcome this difficulty with similar systems include corrections to on-site energies and hopping integrals at the edges of nanoribbons^29^, mainly related to the *π*-bands. Alternatively, an edge potential approach was successfully proposed by Zheng *et al*. as an effective correction to on-site energy values in ZBNNRs^30^. This attempts to explain not only *π*-band features, but also some *σ*-band effects appearing in density functional theory (DFT) results. For the hybrid system *m* BN/*n* G/*m* BN, the presence of a ZGNR in the middle of the boron nitride nanoribbons changes the alternating electron polarization of B and N atoms. In fact, a single edge potential is unable to explain the electronic properties of the hybrid system. For this reason, we look for an electronic effective potential across the BN/graphene interfaces deriving it from DFT results^31^, resulting in on-site energy corrections for our TB calculations. With this parameterization, we study the band gap behavior of hybrid systems as a function of their geometry and structure. We also consider the presence of defects that are usually frequent in such nanostructured systems, mainly at the edges and interfaces, such as edge disorder and atom migration. Spontaneous magnetization in ZBNNRs doped with carbon atoms in substitutional positions in the lattices has been reported^32^, so understanding the effects of such lattice imperfections on the electronic properties is relevant to improve the controlled electronic responses of these nanostructured materials. Actually, atomically controlled substitutional boron doping of graphene nanoribbons has been reported to provide a route for the design of mobility gaps and novel types of graphene transistors^33^.

## Models and Methods

### Hubbard Model

The hybrid system is described by a TB approach involving a set of optimal parameters that properly reproduce DFT calculations. The intrasite electron-electron interaction is included via a Hubbard term, which reproduces the half-metallic characteristics related to spin polarization. The Hubbard Hamiltonian is written as

1 $$ {\mathcal H} =\,\sum _{i\mathrm{=1}}{\varepsilon }_{i}{c}_{i}^{\dagger }{c}_{i}+\,\,\sum _{\langle i,j\rangle }{t}_{ij}{c}_{i}^{\dagger }{c}_{j}+\,\sum _{i\sigma }{U}_{i}{n}_{i\sigma }{n}_{i\bar{\sigma }}+\,{\rm{h}}{\rm{.c}}\mathrm{.,}\,\,$$

where the operator ${c}_{i\sigma }^{\dagger }({c}_{i\sigma })$ creates (destroys) an electron with spin *σ* at site *i*, *n*_*iσ*_ is the spin-dependent occupation number operator, and *ε*_*i*_, *U*_*i*_, *t*_*ij*_ are the on-site energy, the intra-atomic Coulomb repulsion, and integral hopping energy, respectively. Using the mean-field approximation, the Coulomb term in (8) reduces to ${n}_{i,\uparrow }{n}_{i,\downarrow }\approx \langle {n}_{i,\downarrow }\rangle {n}_{i,\uparrow }+\langle {n}_{i,\uparrow }\rangle {n}_{i,\downarrow }$, and the on-site energy may be written as

2 $${\varepsilon }_{i}^{\prime} \equiv {\varepsilon }_{i}+{U}_{i}(\langle {n}_{i,\bar{\sigma }}\rangle -\frac{1}{2}),$$

where the constant factor $\frac{1}{2}$ shifts the band center to $\varepsilon =0$^34^. Charge-neutrality condition is imposed, involving a self-consistent determination of the Fermi level energy. In this process, the average densities $\langle {n}_{i\sigma }\rangle$ were calculated following a real-space renormalization procedure within the Green function formalism, which has been successfully employed to describe different carbon nanomaterials^15,35–37^. Finally, the new on-site energies are calculated from the converged occupation numbers, according to Eq. (2). From these, the final band structures are obtained. A portion of the pristine hybrid system is shown in Fig. 1. A pair of two dimer lines constitutes a translational unit cell, that we denominate an armchair chain. Hence, Fig. 1 shows a 2-armchair-chain supercell. The nanoribbon has translational symmetry along the longitudinal axis, parallel to the zigzag edges. Therefore, we use the same *U*_*C*_ (*U*_*B*_, *U*_*N*_) values for carbon (boron, nitrogen) atoms along the length of the nanoribbon. For non-pristine systems, we consider periodic defects by taking a defective supercell, and following the same self-consistent renormalization procedure described above.

### DFT calculations

In order to obtain the Hubbard and TB parameters, we follow a similar procedure used for other h-BN/graphene systems^38^. We use the Quantum ESPRESSO (QE)^39^ code to compute the electronic structure for the hybrid systems, followed by the WanT Package^40,41^ to obtain the on-site and hopping parameters. We call them the post-processed DFT parameter sets. Pseudopotentials from the ultrasoft Vanderbilt type, a cutoff energy of 60Ry, and a k-point sampling for the zigzag nanoribbons of 52 points are employed.

The codes provide a procedure based on projecting the Kohn-Sham solutions into localized atomic orbitals. The corresponding Hamiltonian in the real space has a Slater-Koster-matrix shape^42^ from which we obtain the TB parameter values. Although post-processing DFT calculations consider interactions between a large range of neighbor atoms, we are just interested in the on-site energy and first nearest-neighbor hoppings, so parameters obtained from QE and WanT are adjusted to build an optimal set of TB parameters which give the best agreement to the DFT calculations.

Optimal on-site energies and hopping parameter values for other BN/graphene systems are reported elsewhere^5^. For the present hybrid system, we adjust those parameter values to obtain an electronic effective potential^31^, affected by the presence of ZBNNR’s edges and BN/C interfaces. More detailed description on the optimized tight-binding model used is presented as a Supplementary Material.

## Results

### Edge and interface potentials

Taking into account the important contribution from edge states to the electronic density of zigzag BN nanoribbons and the electronegativity difference between B and N atoms, Zheng *et al*. proposed a TB model for a ZBNNR system with on-site energy corrections in the form of effective edge potentials^30^. Here we follow a similar approach. In addition to the edge corrections, we also consider the effects of the zigzag interfaces (N/C and C/B), on the on-site energies of the hybrid system.

**Figure 2:**
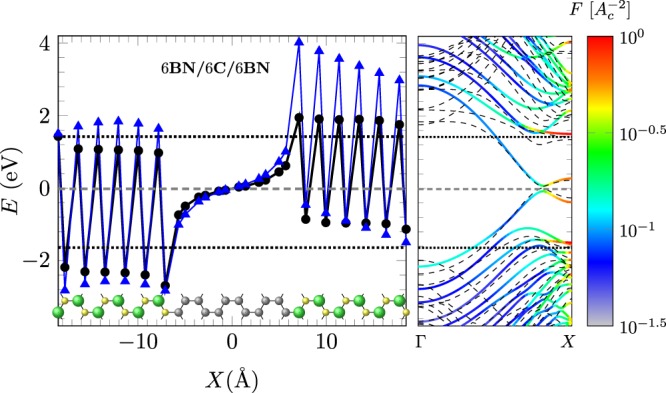
6BN/6 G/6BN hybrid system, with the unit cell shown at the bottom. Left: On-site energies from post-processing DFT calculations (black circles), and the optimal ones (blue triangles) which achieved the best agreement between DFT and TB calculations. Right: DFT (dashed lines) and TB (colored lines) bands of the hybrid system. Colors indicate the degree of localization according to Eq. 6 (*A*_*C*_ is the area of the unit cell). Dotted lines match the effective on-site energy values (B and N), evaluated at the left and right interfaces, respectively, according to Eq. 3. It is noteworthy the correspondence with the localized bands (B and N). The gray horizontal dashed line in the center indicates the Fermi level.

The results obtained from post-processing DFT calculations for the on-site energy values, *ε*_*i*_, for B, N and C atoms are shown as black circles in Fig. 2 (left panel), which may be fitted into an envelope function according to

3 $${\varepsilon }_{i}={\varepsilon }_{i}^{\mathrm{(0)}}+V({x}_{i}),$$

where ${\varepsilon }_{i}^{\mathrm{(0)}}$ are the on-site energies for the *i*-sites far enough from the edges or interfaces.

The envelope function *V(x)* is not directly obtained from post-processing DFT calculations due to the influence of a large number of neighbor interactions. Here, we are interested in an *effective* TB model only considering first nearest-neighbor interactions. Blue triangles in the left panel of Fig. 2 are obtained by using an optimal envelope function that reproduces quite well the band structures of several hybrid systems. This function is obtained following the approach by Zheng *et al*. to correct the on-site energies in ZBNNRs using exponential-type edge potentials^30^. Furthermore, our procedure incorporates the effects from BN edges and BN/graphene interfaces, i.e., for the BN region we have the following contribution from the edges,

4 $${V}_{{\rm{edge}}}(x)={P}_{B}{e}^{-|x-{x}_{B}|/\lambda }+{P}_{N}{e}^{-|x-{x}_{N}|/\lambda },$$

which accounts for the different electronegativities and high *π*-electron density at *x*_*B*_ and *x*_*N*_ edges, while in the internal graphene strip the contributions from the interfaces are chosen as decaying exponential functions centered at the left and right interfaces, *x*_*L*_ and *x*_*R*_,

5 $${V}_{{\rm{int}}}(x)=-\,{P}_{G}{e}^{-|x-{x}_{L}|/{\lambda }_{G}}+{P}_{G}{e}^{-|x-{x}_{R}|/{\lambda }_{G}}\mathrm{.}$$

On-site energies taken directly from post-processing DFT calculations do not reproduce some features of the band structures due to the contributions of many neighbors and the poor projection of Kohn-Sham solutions over localized atomic orbitals^43^. In order to identify the edge-localized states in a band, we compute the analogous of the inverse participation number for the tight-binding coefficients of the wavefunction, which quantifies the localization of a state^44,45^. Thus, we estimate the degree of localization in every band according to

6 $$F\equiv \sum _{i\mathrm{=1}}^{N}{|{c}_{i}|}^{4}\,\,,$$

where the ${c}_{i}$’s are the probability amplitudes of the electronic eigenfunction for the orbital $|{\varphi }_{i}\rangle$ centered at site $i$, in an $N$-atom unit cell. By recognizing the edge states in a band structure, we can compute the corresponding B and N on-site energies by performing a fit to those bands.

The band structures are shown in the right panel of Fig. 2 for both DFT and TB calculations. For the TB bands, the degree of localization is indicated in color, with a maximum value of $F=1$ when the electron is localized in just one atom, corresponding to B or N atoms at the edges of the hybrid zigzag nanoribbon unit cell (dotted lines). By adjusting those on-site energy values, we are able to match the boron-nitride edge states of DFT and TB band structures.

The resulting parameters for edge and interface potentials are shown in Table 1. ${\lambda }_{G}$ describes a short-range interface potential when compared to the edge potential ranges, $\lambda$, in the BN nanoribbon regions. This is reasonable since a graphene strip is only composed of carbon atoms, for which charge carriers are more susceptible to electronic screening compared to the BN region.

**Table 1:** Parameters for the edge and interface potentials.

*P*_*B*_	*P*_*N*_	*P*_*G*_	*λ*	*λ*_*G*_
−1.18 eV	−0.66 eV	−1.50 eV	9.0Å	1.7Å

**Figure 3:**
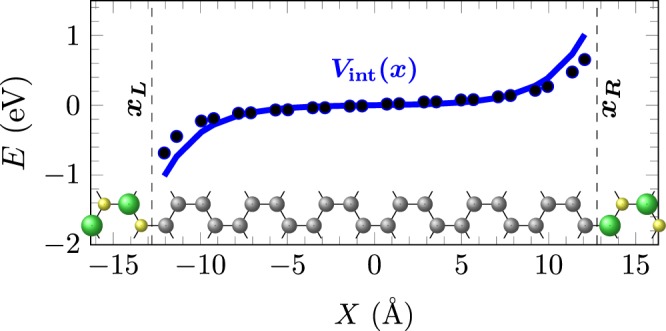
2BN/12 G/2BN hybrid system, with the unit cell shown at the bottom. Black circles indicate on-site energy values from post-processing DFT calculations, while the blue line is the exponential-type fitting proposed, ${V}_{{\rm{int}}}(x)$.

It is important to remark that the same TB parameters explain the main band structure features of other hybrid systems. Actually, we have worked with a set of $m$ BN/$n$ G/$m$ BN such as $(m;n)=\mathrm{(5;2),\; (5;3)...(5;6),\; (3;6),\; (4;6),}$ $\mathrm{(4;4),\; (2;12)}$, and the interface potential proposed fits them even for wider graphene strips, as shown in Fig. 3, where the exponential behavior from interfaces are more clear.

**Figure 4:**
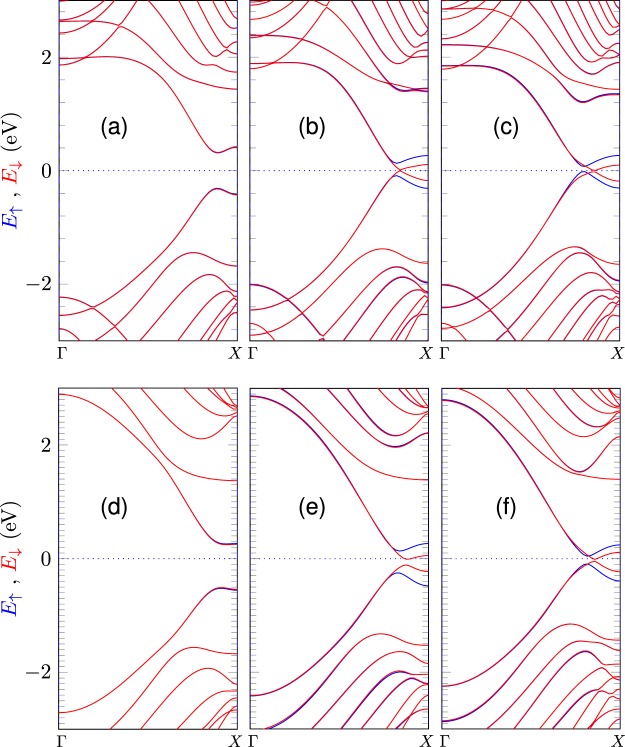
DFT (top) and TB (bottom) band structure calculations for *n* = 2 (left), 4 (center), and 6 (right) of 5BN/*n* G/5BN hybrid systems. Dotted lines indicate the Fermi level. Blue and red curves correspond to up and down bands, respectively.

The curves were fitted by taking ${\varepsilon }_{i}^{\mathrm{(0)}}$ values in agreement with results from literature for other heterostructures made up of graphene and h-BN^5,46^. The work by Zhao *et al*. provides TB parameters for BN quantum dots embedded in graphene^5^. Starting from those values, we have modified them (see Table 2) to better reproduce the DFT band structures of the hybrid systems. Importantly, we depart $\mathrm{[0.10]}eV$ from their ${t}_{CN}$ value to ensure non-magnetic solutions for $5$ BN/$2$ G/$5$ BN, as predicted by DFT results (see Fig. 4(a)).

**Table 2:** Optimal on-site and hopping parameters for the zigzag hybrid system.

${\varepsilon }_{C}^{\mathrm{(0)}}$	${\varepsilon }_{B}^{\mathrm{(0)}}$	${\varepsilon }_{N}^{\mathrm{(0)}}$	*t*_*cc*_	*t*_*CB*_	*t*_*CN*_	*t*_*BN*_	*U*_*C*_	*U*_*B*_	*U*_*N*_
0.0	3.31	−1.09	−2.65	−2.25	−1.80	−2.40	2.7	0.0	0.0

We include Hubbard potential values. Energy units are in eV.

An interesting result of the hybrid systems is their spontaneous half-metallicity without the need of an external field, which is explained by the presence of an effective electric potential generated through the graphene strip, as it can be noticed from Eq. 5. If the width of the graphene nanoribbon is comparable to *λ*_*G*_, the interface potential resembles the potential of a uniform and transversal electric field in a graphene nanoribbon that induces half-metallic behavior above a critical electric field^13^. Particularly, as shown in Fig. 4 (top panels), our DFT results predict a non-magnetic solution for the shortest nanoribbon *m* BN/*n* G/*m* BN with (*m*;*n*) = (5;2), but predicts a half-metallic response for wider ribbons, with (*m*;*n*) = (5;4) and (5;6), in agreement with Ding *et al*.^4^. Similar band structures with half-metallic response are also exhibited by other h-BN/graphene configurations^47^. Tight-binding band structures are also calculated with our parameterization and compared with the DFT calculations. The TB results are plotted in Fig. 4(d–f), displaying a quite similar behavior for edge states and valence bands. Additionally, the TB results present the the same trend of closing the gap shown by the DFT results for wider graphene strips.

### Applying an external electric field

In order to further explore the half-metallicity features observed in the hybrid graphene/h-BN nanoribbons we consider the case in which an extra electric field is applied in the transversal direction of the nanoribbons. To introduce the electric field into the calculations, we have followed the same procedure as in Wakabayashi *et al*.^34^, i.e. the on-site energies are modified by an electrostatic potential:

7 $${\varepsilon }_{i}^{^{\prime} }={\varepsilon }_{i}+V(x),$$

where *V* is an electrostatic potential produced by a transversal electric field, parallel to axis *X* of the figures shown in this article and perpendicular to the edges

8 $$V(x)=x{E}_{field},$$

with *V* being antisymmetric respect to the axis of the nanoribbon. We have chosen this model since our system is electrically neutral, and our calculations follow a procedure that ensures charge neutrality for the system as a whole.

In the presence of a transversal electric field, first-principle calculations on zigzag BNNRs are shown to exhibit asymmetric responses^48^. Such behavior can be understood due to the absence of electron-hole symmetry.

**Figure 5:**
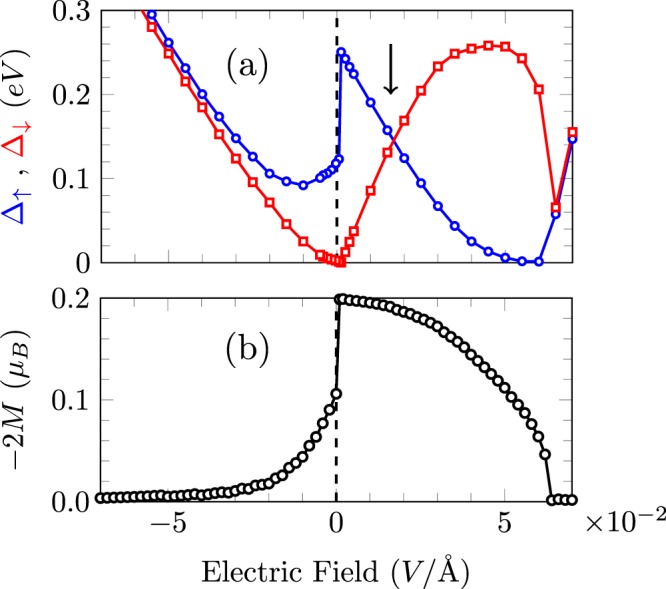
(**a**) TB calculations showing the spin-dependent gap response with respect to an external electric field and (**b**) magnetization of carbon atoms at the left interface BN/graphene of a 5BN/6 G/5BN system.

TB calculations show that when adding a positive external electric field on the hybrid system, the original half-metallicity disappears; with increasing electric field, the bandgap for the spin-up channel decreases rapidly, while the bandgap for the spin-down channel widens. The bandgaps for the up and down spin channels are eventually equal at a particular electric field, marked in Fig. 5(a) by an arrow. Further increasing the electric field, the dependence of the spin up and down gaps with the applied electric field resembles that of a pristine ZGNR. Eventually, it reaches the critical electric field at which the system exhibits half-metallic behavior^3,49^. After this point, the system becomes a nonmagnetic semiconductor. In other words, in the hybrid system, a first critical field occurs when a nonzero external field is applied (removing the original half-metallicity), and a second one happens for larger values, marking the occurrence of the half-metallic behavior. This second critical electric field is shifted to higher values compared to pure graphene nanoribbons, due to the intrinsic field produced by the ZBNNR edges.

According to our TB calculations, the rate of change of the spin-up gap with respect to the electric field is very high around the first critical electric field value (next to zero field for h-BN/graphene heterostructure). We have checked that the exact electric field for which this abrupt transition takes place depends on the Coulomb potential *U* adopted, but the shape of the gap curve as a function of the electric field is completely preserved (more details are presented in the Supplementary Material). TB calculations from Culchac *et al*. also show a fast evolution of the gap value around the critical electric field value for ZGNRs^49^. The rate of change is smoother according to DFT calculations on ZGNRs, but strongly depends on the functional employed^14,50^. It is important to remark that the TB calculations can reproduce the removal of half-metallicity for large values of the electric field, a common feature for ZGNRs and h-BN/graphene heterostructures. For negative values of the electric field, the different spin-dependent gaps are moved towards a non-magnetic solution, with degenerate up and down solutions. Therefore, the addition of an external electric field breaks the intrinsic half-metallic behavior that happens for certain hybrid system geometries. This response is in agreement with the DFT results by Bohwmick *et al*.^20^.

We also calculate the magnetization of the hybrid system 5BN/6 G/5BN as a function of the applied electric field. We use the expression ${M}_{i}=({n}_{i,up}-{n}_{i,dw}\mathrm{)/2}$ for the local magnetization at site *i*, given in terms of the Bohr magneton, with *n*_*i,up*_ (*n*_*i,dn*_) being the mean number of electrons with spin up (down) at site *i*. The results plotted in Fig. 5(b) clearly show a magnetization quenching for large electric-field values (both directions). This response also resembles the magnetization behavior of a zigzag graphene nanoribbon in the neighborhood of the critical value of the external transverse electric field^49,51^. A positive external field increasing also leads to a magnetization quenching, but with different field-dependence due to the internal electric field induced by the BN edges. Of course, the critical electric field which produces the magnetization suppression depends on the characteristic sizes of the hybrid system, that may be additionally engineered.

### Substitutional defects: roughness and diffusion in hybrid nanoribbons

**Figure 6:**
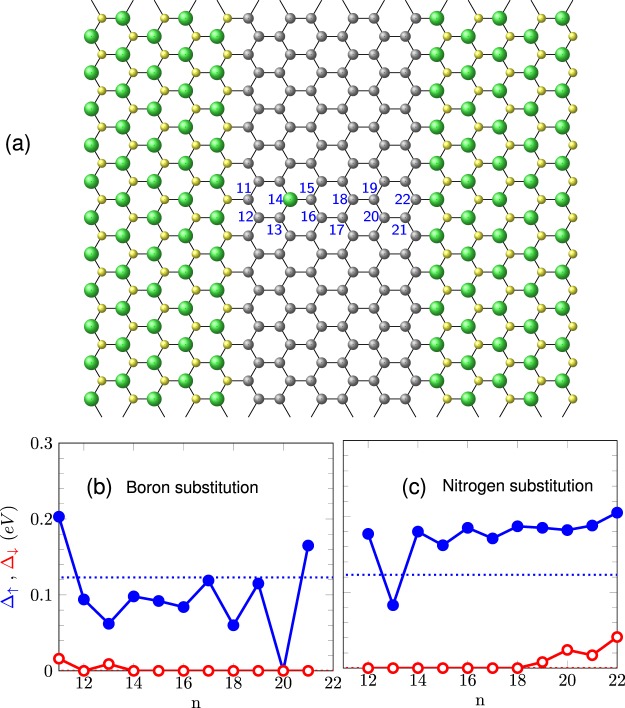
Impurity substitutions along the zigzag graphene ribbon in a supercell of 11 armchair chains shown in (**a**). A boron atom substitutes a carbon atom at the *n*-th position (shown by a green ball at *n* = 14). Panels (**b**,**c**) show the gap evolution with respect to the substitutions at the *n*-th position for the B and N cases, respectively. Filled blue (red) dots correspond to the spin up (down) gaps. For comparison, the bandgaps of the pristine systems with perfect interfaces are shown with dotted lines, blue and red denoting up and down channels, respectively.

A more realistic description of the studied hybrid nanoribbons should certainly include roughness in the zigzag interfaces as well as possible diffusion of boron or nitrogen atoms from the h-BN region into the graphene strip^52^ and vice versa. The presence of defects at the interface has been reported elsewhere^53^ and could lead to an enhancement of the half-metallic behavior^54^. A DFT description of these hybrids would require high computational cost. Dieb *et al*. overcame this difficulty^55^ proposing a machine learning algorithm to describe B-doped graphene systems and to find the most stable configurations. Alternatively, we model hybrid systems by means of the tight-binding approximation, using our TB parameterization and fitting curves to model the hybrid system bandgaps. We first consider the possibility of boron or nitrogen atoms placed at carbon atom positions inside the graphene strip, mimicking a migration process, as shown schematically in Fig. 6(a). Notice that the considered supercell with a single substitutional B or N atom is repeated along the edge direction. This can be described following a real-space renormalization scheme.

We analyze the gap evolution shown in Fig. 6(b,c), with respect to the B and N substitution at the *n*-th site. We should remember that nitrogen electronegativity is higher than those of carbon and boron atoms. Usually, in an B-doped AGNR the Fermi level is shifted downward due to missing *π* electrons from the B atoms^56^. As expected, impurity substitutions with boron and nitrogen atoms induce opposite trends for the bandgap behavior as a function of the impurity position along the graphene material: averaged higher/smaller spin-up gap as the B/N atom migrates from the N-C (left) to the C-B (right) interface. N-substitutions along the graphene strip show, on average, a more robust half-metallic response compared with B-substitutions and to the pristine case. Otherwise, notice that the system enhances the half-metallic response when B and N substitutions happens at the first neighbor carbon atom in both interfaces; site 11 for the B and site 22 for the N impurity. When a B occupies the 11-th position, it follows the natural B-N sequence along the h-BN armchair chain, leading to a narrower effective ZGNR width, what makes the gap increase. The same is valid for an N impurity at site 22. Calculations for less diluted systems (one impurity per supercells of 3, 5 and 7 armchair chains) reveal similar bandgap trends, but with more variations.

As we have not directly included the structural distortions in the discussion on the defects, we have explored a possible alternative approach to include effectively the lattice distortion effects, based on the linear strain theory that changes hopping energies within tight-binding descriptions. Details are presented in the Supplementary Material. We have shown that similar results are obtained, validating our approach.

Using our TB parameters with edge and interface potentials, we calculate the total energy of the system when a boron (or a nitrogen) atom substitutes a carbon atom in the graphene strip. The results show (not presented here) that a boron replacing a carbon atom at the interface is the most favorable energetic configuration, where the boron is bonded with a nitrogen atom. As expected, an analogous behavior happens for the nitrogen substitution on the other edge. These results have been corroborated by DFT calculations.

**Figure 7:**
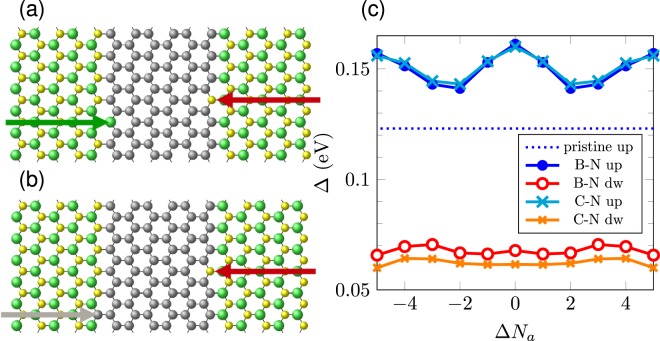
Schematic view of part of a supercell with a pair of impurities (**a**) B-N and (**b**) C-N. The red arrows indicate the N impurity and the green (gray) arrow marks the B (C) impurity. The B (C) impurity migrates vertically along the left interface occupying the *N*_*a*_-th armchair chain. The N impurity substitutes a carbon atom at the right interface on the 0-th armchair chain. For Δ*N*_*a*_ = 0, the two impurities belong to the same armchair chain, located at each interface. (**c**) Gap behavior with respect to the relative position Δ*N*_*a*_ of the two impurities B-N or C-N. The results refer to an 11-armchair chain supercell.

We also study other defect configurations. Instead of having atomic diffusion across the graphene strip, we analyze the effect of the presence of impurities located at opposite interfaces. Figure 7(a) shows an example of defects at both h-BN/graphene interfaces; i.e., a nitrogen atom is located at the B/C right interface, substituting a carbon atom, while a boron atom is located at the N/C left interface in the graphene strip. A similar framework is considered in Fig. 7(b) with a carbon impurity substituting a nitrogen atom at the N/C left interface, maintaining the nitrogen impurity fixed at the right interface (red arrow). The effect on the energy bandgap due to the relative position of the defects is displayed in Fig. 7(c). We define Δ*N*_*a*_ as a parameter that indicates how far the two defects are from the frontal relative position, in terms of the number of armchair chains separating the two defects. Thus, Δ*N*_*a*_ = 0 when both impurities belong to the same armchair chain, but located at different interfaces.

Figure 7(c) shows two main features: an oscillatory gap behavior with respect to the relative impurity positions at the opposite interfaces for the up component, and a nearly constant energy gap for the down component. The former can be explained in terms of the modification of the interface potentials due to the presence of impurities at each interface. The effective field generated by the charged boron and nitrogen atoms at the interface is perturbed in the presence of defects at the interfaces, which leads to a change on the bandgap of the system. Figure 7(c) shows an overall increase for the bandgap values respect to pristine case. The half-metallic character is destroyed; both up and down spin-gaps are finite. Moreover, Fig. 7(c) shows a maximum gap value when the impurities are exactly in front of each other, i.e., when Δ*N*_*a*_ = 0, while for other neighboring relative positions of the defects, the gap starts decreasing as the impurities get apart from each other. In fact, the configuration with opposite impurities amounts to an effective reduction of the width of the ribbon, which yields an increased gap.

**Figure 8:**
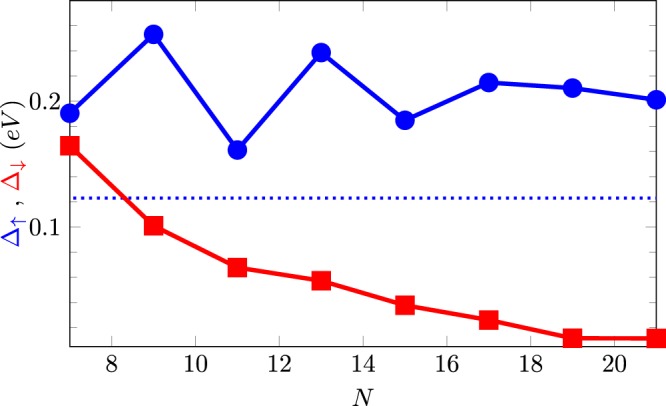
Spin-up and down gaps as functions of the supercell size *N* for a $5BN\mathrm{/6}G\mathrm{/5}BN$ system with impurities in the same armchair dimer line. For comparison, the bandgap of the pristine system with perfect interfaces is shown with a dotted line.

We should also keep in mind that the oscillatory gap behavior as a function of Δ*N*_*a*_ shown in Fig. 7(a,b) is a direct consequence of the translational periodicity imposed by the repetition of the supercell containing the two impurities. We have obtained the same oscillatory trend for other supercell sizes. Additionally, we have analyzed the role of the supercell size in the calculations. Figure 8 shows the dependence of the spin-up and down gaps as functions of the supercell size *N* for the particular system $5BN\mathrm{/6}G\mathrm{/5}BN$. The system tends in the dilute impurity limit to a half-metallic configuration, with spin-down gap tending to zero for larger sizes, though still showing a sizable spin-up gap, as in Fig. 7, but the overall behavior is well described with smaller supercells. More systematic studies are necessary, however, considering for instance the effect of different impurity concentrations at the edges on the electronic properties of h-BN/graphene heterostructures, and also doping the graphene strip region. Miao *et al*. reported^57^ that doping graphene sheets above a certain concentration leads to a half-metallic response. Therefore, the combined effects of interface potentials and a B(N)-doping in h-BN/graphene/h-BN systems may result in an enhancement of the half-metallic behavior depending on the doping concentration.

### Final Remarks

In summary, we have found TB parameters for the *n* BN/*m* C/*n* BN heterostructure that reproduce the main features of DFT predictions including the effect of external electric fields. The TB parameters are not only valid for a specific size of the heterostructure: they are robust enough to explain main trends of the electronic band structures for a wide range of graphene/h-BN system sizes. Due to the ionic edges of ZBNNRs and BN/C interfaces in the hybrid systems described here, we can estimate an effective potential that gives a best fit to DFT predictions. Another advantage of our TB parameters is the study of transport using Green functions in big hybrid systems and the influence of impurities in a scattering region. The coupling regions between electrodes and scattering regions are minimized, reducing the computational cost of the transport calculations. We have enlarged our discussion on the adequateness of our approach and the possibility of validating our approach with other computational methods in the Supplementary Material.

With respect to the applied electric fields, due to the polarity of the material, boron and nitrogen edges induce an electric field that competes with the external field, that we have successfully modeled. Half-metallicity becomes intrinsic to the BN/5 C/BN even in the absence of an external electric field. Furthermore, the graphene/h-BN heterostructure can not only work as a spin filter, but also as a spin swapping device by just turning on an external electric field.

Taking advantage of our approach, we aim to study larger systems that would be computationally expensive by first-principles calculations. We use our TB parameters to analyze more realistic graphene/h-BN heterostructures by considering boron and nitrogen diffusion across the graphene strip, or graphene/h-BN interface with roughness due to substitutional impurities. We have found that in general nitrogen diffusion enhances the half-metallic response. We have also found an oscillatory gap behavior that is maximum for a pair of boron and nitrogen impurities facing each other at each BN/graphene interfaces. We attributed this maximum gap value to an effective reduction of the width of the nanoribbon. Further studies are needed to analyze a possible suppression or enhancement of the half-metallic response by modulating the concentration of boron and nitrogen impurities at the interfaces.

## Supplementary information

Supplementary Material
