# Supplementary material for: Interface effects in hybrid hBN-graphene nanoribbons
Source: Sci Rep. 2019 Mar 5;9:3508. doi: 10.1038/s41598-019-39763-5 (PMC6401180; doi:10.1038/s41598-019-39763-5)
Supplement: Supplementary file 1 — Supplementary Material [file 41598_2019_39763_MOESM1_ESM.pdf]

Supplementary material: Interface effects in hybrid hBN-graphene nanoribbons

Carlos Leon[1], Marcio Costa[1], Leonor Chico[2,+], and Andrea Latgé[1,+,*]

[1]Instituto de Física, Universidade Federal Fluminense, Niterói, Av. Litoranea sn 24210-340, RJ-Brazil
[2]Instituto de Ciencia de Materiales de Madrid (ICMM), Consejo Superior de Investigaciones Científicas (CSIC), Madrid 28049, Spain
[*]andrea.latge@gmail.com
[+]these authors contributed equally to this work

ABSTRACT

We analyze the electronic properties of a hybrid graphene-BN nanoribbon system, using a Hubbard model Hamiltonian within a mean field approximation. Due to the different electronegativities of the boron and nitrogen atoms, an electric field is induced across the zigzag graphene strip, breaking the spin degeneracy of the electronic band structure. Optimal tight-binding parameters are found from first-principles calculations. Edge potentials are proposed as corrections for the on-site energies, modeling the BN-graphene nanoribbon interfaces. We show that half-metallic responses in the hybrid systems may be driven with the help of an external electric field. We also study the role of defects across the graphene nanoribbon and at the h-BN/graphene interface regions. Modulations on the spin-dependent gaps may be achieved depending on the nature and position of the defect, constituting a way towards spin-gap engineering by means of spatial doping.

Optimized Tight-binding Parameters

Our procedure to find the hopping, on-site values, and the coefficients of the potentials involves an interplay between the effect of those parameters on the TB band structures of several sizes compared to DFT results. A post-processing DFT calculation using QE and WanT returns a Hamiltonian projected in real space[1], with the shape of a Slater-Koster matrix. From here, we can find a reference for the hopping and on-site values. However, it is be necessary to take the TB parameters of at least 5 nearest neighbors to reproduce DFT band structures. Instead, we have taken the WanT results as a starting point, and then studied the effects of each parameter on the TB band structures considering interaction of just first nearest neighbors. As we stated in the manuscript, the parameters found can explain the main features of nanoribbons as narrow as 2-ZGNR stripes and wider as 12-ZGNR in the middle of the hybrid system.

In Figure 1, we show an example of the effect of nearest neighbors given by WanT, and the optimal TB parameters involving just first nearest neighbors that we have found for 2D graphene.

Fig. 2 shows the behavior of the on-site energy along the hybrid system (left), and band structures (right) for two systems, 2BN-12G-2BN(a) and 5BN-2G-5BN(b). In the widest case (12G), we can clearly notice the exponential behavior of the interface potentials. In the narrowest case, the same interface potential is used, and still reproduces quite well the central region

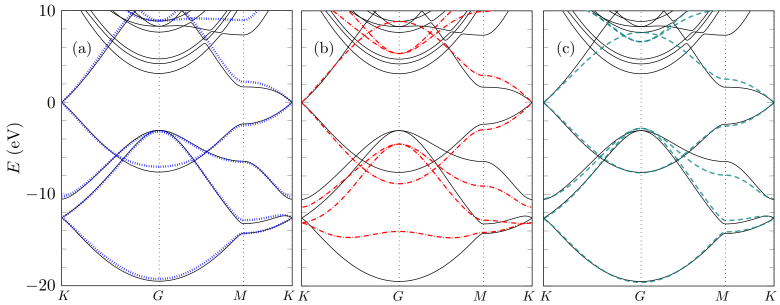

Figure 1. (Color online) Black lines indicate DFT graphene band structure and colored lines correspond to TB calculations. Band structure diagram using (a) 5 first nearest-neighbors from WanT, (b) first-neighbors hoppings from WanT, and (c) an optimal set of fitted hopping values, in blue, red, and green dashed lines, respectively.

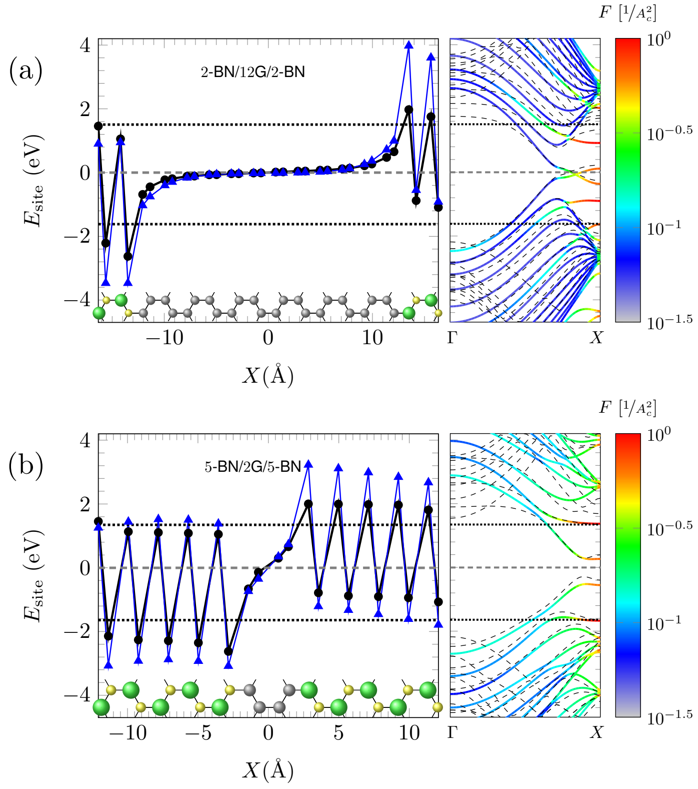

Figure 2. (Color online) Results for (a) 2BN/12G/2BN and (b) 5BN/2G/5BN structures. Left: On-site energies according to DFT calculations (black circles) and TB model (blue triangles). The insets show the corresponding unit cells of the zigzag hybrid systems. Right: DFT (dashed black lines) and TB (colored lines) band structures, where colors indicate the F-localization. The same TB parameters and fitting curves were used for short (top) and long (bottom) graphene stripes. Dotted lines indicate the effective on-site energy values (B and N). The gray horizontal dashed line in the center indicates the Fermi level.

of the hybrid system. The other TB parameters are also fit. Even though it is possible to recognize band structure differences in the localized part of the B-N region, the model agrees with the bandgap of the system (as shown in Figure 4 of the manuscript). Other band structure differences are related to the fact that we are just considering the interaction of first nearest neighbors (as we mentioned earlier, it would be necessary to take at least the 5 nearest neighbors to reproduce fully the DFT band structures).

Electric field effects

We also took into account the effects of an external electric field on the gap evolution to fit the TB parameters. The $U = 2.7\,\text{eV}$ best reproduces DFT results, as it explains half-metallicity at zero electric field. We have compared our TB calculations to DFT results when electric field is present. Bhowmick *et al* show the spin-dependent gap behavior respect to an external electric field (see Figure 5d of Bhowmick *et al*[2]). The gap is spin-polarized at $E_{\text{field}} = 0$. The gap of spin-up channel decreases while the gap of spin-down channel widens for an increasing electric field. Eventually, the gap of both spin-channels crosses each other. This overall gap evolution is similar to our TB results show in Fig. 5(a) of the manuscript for the region between $E_{\text{field}} = 0$ and $E_{\text{field}} \sim 2V/\text{Å}$ (marked with an arrow).

To compare our TB findings with DFT calculations we also included here, in Figure 3, the magnetization evolution of a carbon atom at one interface with respect to an external electric field applied in the hybrid system, calculated by first principle using the VASP package. The magnetization goes to zero for negative electric fields, reaches a maximum value around $E_{\text{field}} = 0$, and then decreases for higher electric fields. This same behavior is found in our TB results shown in Figure 5(b) of the manuscript.

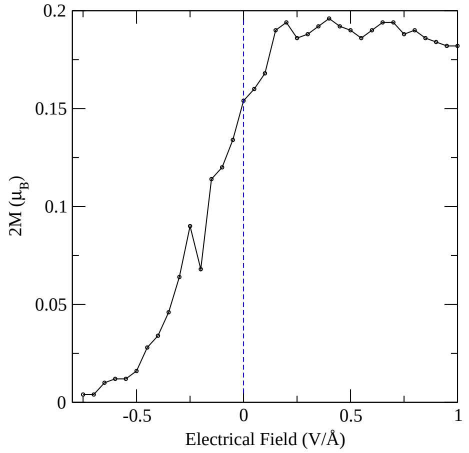

Figure 3. (Color online) Edged carbon-atom magnetization evolution with respect to an external electric field for a hydrogen-passivated 5BN-6G-5BN hybrid system, by first principle calculations.

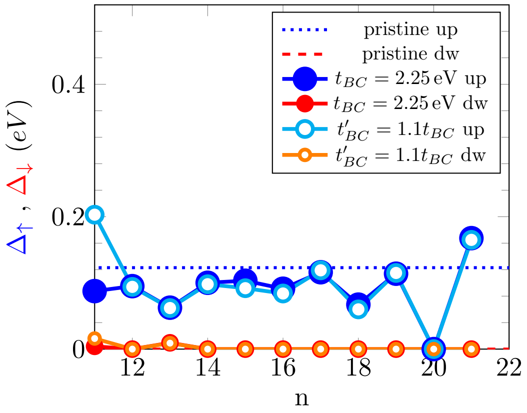

Figure 4. (Color online) Gap evolution respect to position of the carbon substitution by a boron atom for two t_{BC} values. The supercell contains 11 single unit cells of 5BN/6G/5BN.

Distortion effects

We have not directly included the structural distortions in the discussion on the defects. Depending on the relative difference between the host and defect atomic number, the distortions can be important, mainly for very different atomic species, but that it is not our case.

Specifically, carbon substitutions by boron atoms produce energetically favorable in-plane distortions with a B-C bond length of approximately 1.49-1.50 Å[3,4]. Also, in-plane distortions when a nitrogen atom substitutes a carbon atom have a N-C bond length of 1.41Å[5]. Considering then these new values for the bond lengths we may estimate the effective hopping energies due to these distortions, accordingly to the following expression:

$$t' = t_0 \exp -k(l/l_0 - 1), \tag{1}$$

where t_0 is the hopping without perturbation, l_0 is the carbon-impurity distance, and the parameter k is the electromechanical coupling strength given by, $k = |\partial \log t_0 / \partial \log a|$ (where a is the carbon-carbon distance, and in the case of graphene $k \sim 3$). This equation has been currently used in tight-binding descriptions of strained carbon systems[6], within the elasticity theory[7,8].

Following this model the previously addressed distortions in the B-C and N-C bond lengths would lead to associated changes in the nearest-neighbor hopping values around $\sim 10\%$ for boron impurities, and 1% for nitrogen impurities. Figure 4 shows a comparison between the effects of a t_{BC} and a 10% increase on the gap values when a boron atom substitutes a carbon atom in the graphene ribbon. The curves are very similar except at points when the boron atom is more influenced by the interfaces.

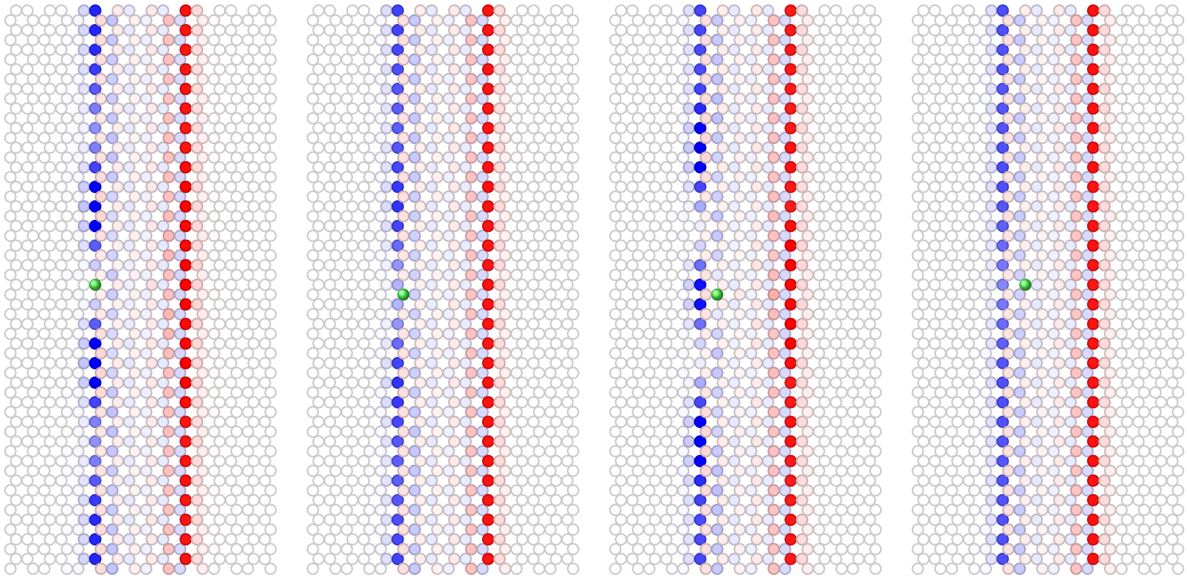

Figure 5. (Color online) Local magnetization through the hybrid system when a boron atom (green ball) substitutes a carbon atom at four different positions. The figures shown are supercells made up of 29 5BN/6G/5BN single lines. The system is periodic along the vertical axis.

Magnetization in larger systems

Single *m*BN/*n*G/*m*BN unit cells and other BNC heterostructures are feasible to work with by means of DFT methods, and also large number of atoms can be tackled with DFTB approaches. Indeed, DFTB could be a way to validate our approach. However, we do feel that reducing the coupling to nearest-neighbor interactions is important when studying, for instance, large systems as shown in Figure 5, with 928 atoms per supercell. Here we study the effect of a diluted concentration of impurities on the magnetization at each atom. Our TB results using the optimal parameters found by our method allow us to predict -at least in a qualitative way compared to DFT calculations- the oscillatory behavior of the magnetization on the interfaces, giving an adequate description of the impurity influence.

References

1. Agapito, L. A., Ferretti, A., Calzolari, A., Curtarolo, S. & Buongiorno Nardelli, M. Effective and accurate representation of extended bloch states on finite hilbert spaces. *Phys. Rev. B* **88**, 165127, DOI: 10.1103/PhysRevB.88.165127 (2013).
2. Bhowmick, S., Singh, A. K. & Yakobson, B. I. Quantum dots and nanoroads of graphene embedded in hexagonal boron nitride. *The J. Phys. Chem. C* **115**, 9889–9893, DOI: 10.1021/jp200671p (2011).
3. Kweon, K. E. & Hwang, G. S. Formation, structure, and bonding of boron-vacancy pairs in graphene: A first-principles study. *Phys. Rev. B* **82**, 195439, DOI: 10.1103/PhysRevB.82.195439 (2010).
4. Faccio, R. *et al.* Electronic and structural distortions in graphene induced by carbon vacancies and boron doping. *The J. Phys. Chem. C* **114**, 18961–18971, DOI: 10.1021/jp106764h (2010).
5. Wang, X. *et al.* Heteroatom-doped graphene materials: syntheses, properties and applications. *Chem. Soc. Rev.* **43**, 7067–7098, DOI: 10.1039/C4CS00141A (2014).
6. Carrillo-Bastos, R. *et al.* Strained fold-assisted transport in graphene systems. *Phys. Rev. B* **94**, 125422, DOI: 10.1103/PhysRevB.94.125422 (2016).
7. Landau, L. & Lifshitz, E. M. *Theory of elasticity. (Volumen 7 of A Course of Theoretical Physics)* (Pergamon Press, Cambridge, 1970).
8. Katsnelson, M. I. *Graphene: Carbon in Two Dimensions* (Cambridge University Press, Cambridge, 2012).
